# Tandem CAR-T cells targeting FOLR1 and MSLN enhance the antitumor effects in ovarian cancer

**DOI:** 10.7150/ijbs.63181

**Published:** 2021-10-22

**Authors:** Zhen Liang, Jiao Dong, Neng Yang, Si-Di Li, Ze-Yu Yang, Rui Huang, Feng-Jie Li, Wen-Ting Wang, Jia-Kui Ren, Jie Lei, Chen Xu, Dan Wang, Yan-Zhou Wang, Zhi-Qing Liang

**Affiliations:** 1 Department of Obstetrics and Gynecology, Southwest Hospital, Army Medical University (Third Military Medical University), Chongqing, China.; 2 Department of Obstetrics and Gynecology, Guangyuan Traditional Chinese Medicine Hospital, Guangyuan, China.; 3 Breast and Thyroid Surgical Department, Chongqing General Hospital, University of Chinese Academy of Sciences, Chongqing, China.; 4 Institute of Pathology and Southwest Cancer Center, Southwest Hospital, Army Medical University (Third Military Medical University), Chongqing, China.; 5 Department of Internal Medicine, Hui Long-Ba Town Hospital, Chongqing, China.; 6 Department of Hepatobiliary Surgery, Xijing Hospital, The Fourth Military Medical University, Xi'an, China.

**Keywords:** ovarian cancer, CAR-T therapy, FOLR1, MSLN, IL-12

## Abstract

Given the heterogeneity of solid tumors, single-target CAR-T cell therapy often leads to recurrence, especially in ovarian cancer (OV). Here, we constructed a Tandem-CAR targeting two antigens with secretory activity (IL-12) to improve the effects of CAR-T cell therapy. Twenty coexpressed upregulated genes were identified from the GEO database, and we found FOLR1 (folate receptor 1) and MSLN (mesothelin) were specifically and highly expressed in cancer tissues and only 11.25% of samples were negative for both antigens. We observed an increased proliferation rate for these three CAR-T cells, and Tandem CAR-T cells could efficiently lyse antigen-positive OV cells *in vitro* and secrete higher levels of cytokines than single-target CAR-T cells. More importantly, *in vivo* experiments indicated that Tandem CAR-T cells markedly decreased tumor volume, exhibited enhanced antitumor activity, and prolonged mouse survival. Furthermore, the infiltration and persistence of T cells in the Tandem-CAR group were higher than those in the MSLN-CAR and Control-T groups but comparable to those in the FOLR1-CAR group. Collectively, this study demonstrated that Tandem CAR-T cells secreting IL-12 could enhance immunotherapeutic effects by reducing tumor antigen escape and increasing T cell functionality, which could be a promising therapeutic strategy for OV and other solid tumors.

## Introduction

Ovarian cancer (OV) is the leading cause of death among female reproductive system malignancies, causing 207,252 deaths worldwide, and up to 313,959 new cases were diagnosed in 2020 1. Currently, the prognosis for most OV patients is still dismal even though there have been great advances in comprehensive therapeutic strategies, and although median survival has been substantially improved, cure rates remain relatively unchanged 2, 3. Nearly 70% of OV patients are diagnosed at a late stage with distant metastases 4. As a result, between 55% and 75% of women who respond to first-line therapy usually experience relapse within two years and lack effective treatment options when relapse does occur 5. Therefore, developing novel and effective treatment strategies for OV is urgently needed.

During the past decades, immunotherapy has produced impressive effects on various tumors, most of which are treated with immune checkpoint inhibitors such as monoclonal antibodies to eliminate cancer cells via activation of the immune system 6, 7. However, even though this endogenous immune response can avoid some side effects, it has the problem of a low response rate. For example, the KEYNOTE-100 trial showed that the response rate of the anti-PD1 antibody Keytruda in recurrent OV was only 8% 8. To achieve better effectiveness, engineered T cells with a chimeric antigen receptor (CAR) have become a potent treatment capable of efficiently recognizing and exerting cytotoxicity against cancer cells expressing specific antigens 9. To date, CAR-T cell therapy has produced significant benefits in hematologic malignancies that are refractory to traditional treatment but has not yet shown widespread benefits in solid tumors, mostly due to the immunosuppressive microenvironment and immune escape resulting from antigen heterogeneity 10. Marofi *et al* described these mechanisms in detail, including the recurrence of antigen-negative cells, inadequate trafficking and infiltration of T cells, T cell inhibition, and/or insufficient T cell activation in tumor tissue 11.

Facing these obstacles, emerging data suggest that targeting two antigens simultaneously may improve the antitumor activity of CAR-T cells by increasing antigen coverage 12, 13. Hegde *et al* reported that a bispecific CAR molecule (Tandem-CAR) targeting HER2 and IL13Rα2 could reduce antigen escape and achieve sustained tumor elimination in glioblastoma 14. In addition, previous studies have shown that IL-12, which is produced by activated lymphocytes, could enhance the proliferation and survival of T cells, which can induce the cytotoxic response of CTLs and NK cells and increase the secretion of cytokines such as TNF-α and IFN-γ to recruit additional immune cells into the response to eliminate tumor cells not recognized by CAR-T cells 15, 16.

Herein, using bioinformatics and clinical samples, we identified two candidate proteins, folate receptor 1 (FOLR1) and mesothelin (MSLN), that were highly expressed in tumor tissues but exhibited low expression in normal tissues. Specifically, there is a 48% to 76% probability of near-completely eliminating tumor by targeting FOLR1 or MSLN alone, while targeting FOLR1 and MSLN simultaneously was predicted to kill more than 88% of cancer cells based on a cohort of 160 OVs. Thus, we designed a novel Tandem-CAR encoding an anti-FOLR1 scFv, an anti-MSLN scFv, and two peptide sequences of IL-12 to improve the efficacy, infiltration, persistence, and proliferation of CAR-T cells in OV.

## Methods

### Identification of upregulated genes from the GEO database

**Figure 1:**
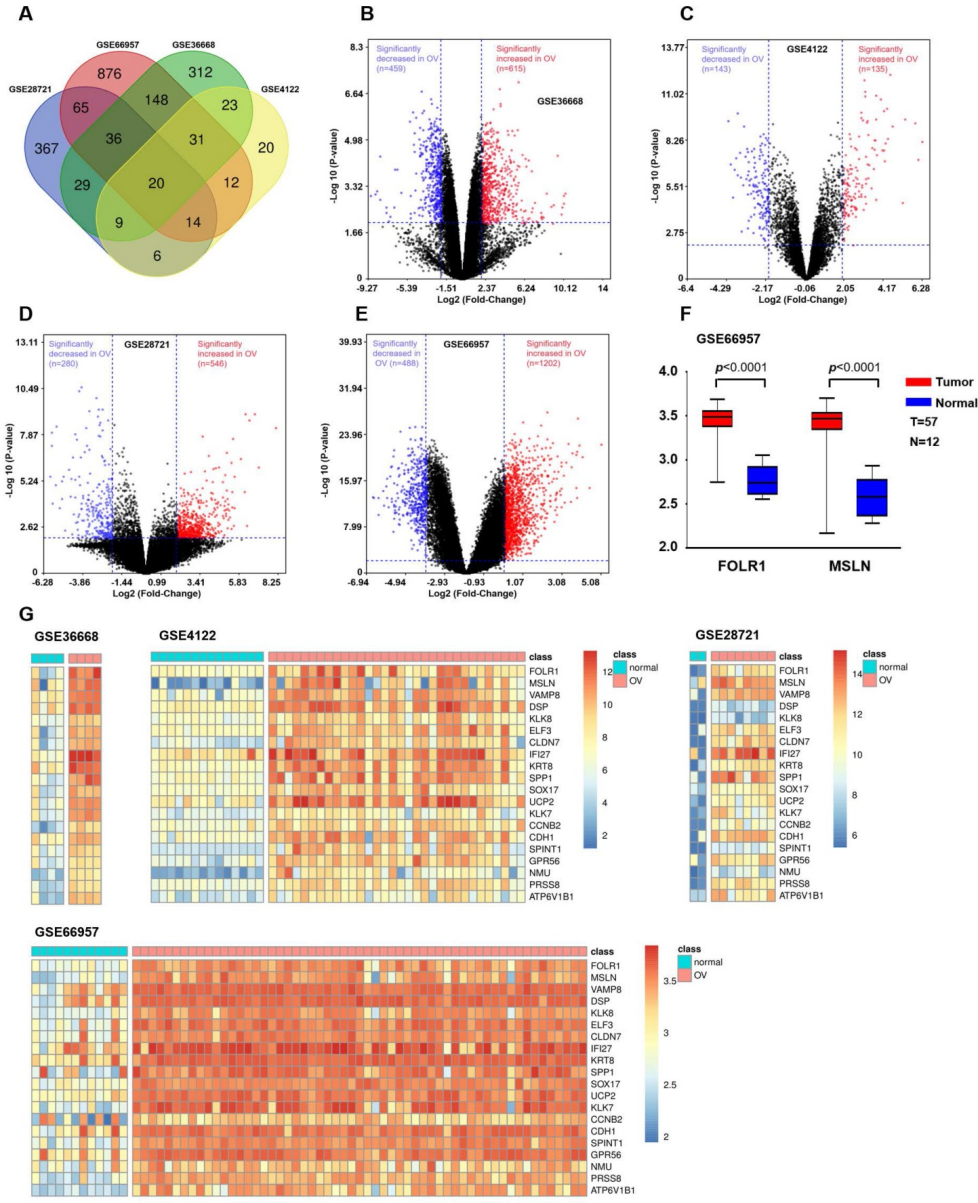
** Bioinformatic analysis to identify candidate genes in OV.** (A) Venn diagram depicting the 20 overlapping upregulated genes from four gene expression microarray datasets (GSE28721, GSE66957, GSE36668, and GSE4122). (B) Volcano plot of GSE36668. The dark red and dark blue dots denote the significantly up- and downregulated genes in OV tissues compared to normal tissues, respectively (*p* value < 0.01 and |log FC|≥2, Student's t-test). (C) Volcano plot of GSE4122. (D) Volcano plot of GSE28721. (E) Volcano plot of GSE66957. (F) In the GSE66957 dataset, elevated expression of FOLR1/MSLN at the mRNA level was observed in OV tissues compared to normal tissues. (G) Heatmap representing the 20 overlapping upregulated genes from different datasets. Red and green indicate high and low expression of the gene in the sample, respectively.

We integrated and analyzed four GEO databases (GSE28721, GSE66957, GSE36668, and GSE4122, Table S1) to identify differentially upregulated genes between OV and normal ovarian tissues (Supplemental Methods). The results are shown in Figure 1. Since tumor-associated antigens and membrane proteins that are specifically expressed on the surface of the cell membrane can be potential targets for immunotherapy, we screened two candidate genes (FOLR1 and MSLN) based on the results obtained from the Venn diagram and heatmap. Then, the mRNA expression levels of FOLR1 and MSLN were compared between tumor and normal tissues in the GSE66957 dataset (n=69).

### Clinical samples

In the present study, two ovarian tissue microarrays were obtained from the National Biological Sample Resource Bank in Shanghai, and each microarray consisted of 158 OV samples and 2 borderline OV samples. The patients from whom these gynecological malignancies were collected underwent surgical procedures between February 1, 2009, and February 28, 2013. Additionally, normal ovary samples (n=25) were obtained from patients with myoma of the uterus and then embedded in paraffin (Avilabio Biotechnology, China). These patients underwent oophorectomy or hysterectomy in the Department of Obstetrics and Gynecology between January 1, 2017, and June 1, 2019. The clinicopathological information of the 160 OV tissue microarray samples is summarized in Table S2.

### Immunohistochemistry staining (IHC) and quantification

IHC staining was conducted as previously described 17, and we performed a quantitative analysis of the intensity of FOLR1- and MSLN-positive staining in all samples to evaluate the expression differences of the two proteins 18 (Supplemental Methods).

### Lentiviral vector production and CAR-T Cell production

The anti-FOLR1 scFv and anti-MSLN scFv fragments used originated from MOv19 scFv 19, 20 and P4-scFv 21, respectively. CAR constructs were synthesized and cloned into the pCDH lentiviral plasmid backbone with a human CMV promoter. A lentiviral vector containing a CAR consisting of the anti-FOLR1 scFv (or anti-MSLN scFv), CD8 hinge region, CD8 transmembrane domain, CD28 and 4-1BB costimulatory signaling molecules, CD3ζ signaling endodomains, and two subunits of interleukin-12 that were linked sequentially was transduced into HEK293T cells. The Tandem-CARs incorporated the scFv sequence of anti-FOLR1 or anti-MSLN through a linker (Supplemental Methods).

### Laboratory assays

We performed flow cytometry, cytokine release, cell proliferation assays, cytotoxicity assay, and xenograft mouse model to analyze all samples. The details were described in the Supplemental Methods.

### Statistical analysis

Quantitative results are presented as the mean ± standard deviation. Analyses of the associations between tumor-associated antigen expression in tissue sections and available clinicopathological factors were performed using the χ^2^ test or Fisher's exact test. Statistical tests were performed with Prism GraphPad 8.0 or SPSS v.22.0 (Chicago, IL, USA). Unpaired Student's *t*-tests, one-way ANOVA, or two-way ANOVA with Bonferroni's post hoc test were used when appropriate. Survival was analyzed with the log-rank test. In all tests, a two-sided *P*<0.05 was considered to be statistically significant.

## Results

### Identification of coexpressed upregulated genes

OV expression profile datasets (GSE28721, GSE66957, GSE36668, and GSE4122) were normalized, and the results of volcano plots indicated that a total of 546, 1202, 615, and 135 upregulated genes were screened from the four datasets, respectively (Figure 1B, C, D, and E). A total of 20 coexpressed upregulated genes from these four gene expression profiles were identified by Venn diagram analysis (Figure 1A). The results of a heatmap indicated that the expression of these 20 upregulated genes differed between the normal and OV tissues in each of the datasets (Figure 1G, H, I, and J). The criterion was genes encoding tumor-associated antigens (TAAs) and membrane proteins, which may be highly and selectively expressed on the tumor surface. Based on the above results, we selected two proteins with higher expression levels in OV: FOLR1 and MSLN. The results for the GEO database data (GSE66957) also indicated that the expression of FOLR1 and MSLN was significantly higher in OV tissues than in normal tissues (*p* < 0.0001, Figure 1F).

### FOLR1 and MSLN were highly expressed in human OV tissues and associated with clinicopathological features

To further investigate the roles of FOLR1 and MSLN in patients with OV, we explored the relationships between FOLR1 or MSLN expression and clinicopathological factors at the protein level, as assessed by IHC staining. The clinicopathological variables included in the present study are listed in Table S2, and the associations between FOLR1 or MSLN expression and clinicopathological criteria are shown in Table S3-S5. As expected, the majority of patients presented with late-stage (stage III/IV) (70.88%) disease and experienced recurrence (80.38%). Forty-six patients had stage I/II disease, 112 had stage III/IV disease, and most patients had serous histology (G3; 65.82%, Table S2).

**Figure 2:**
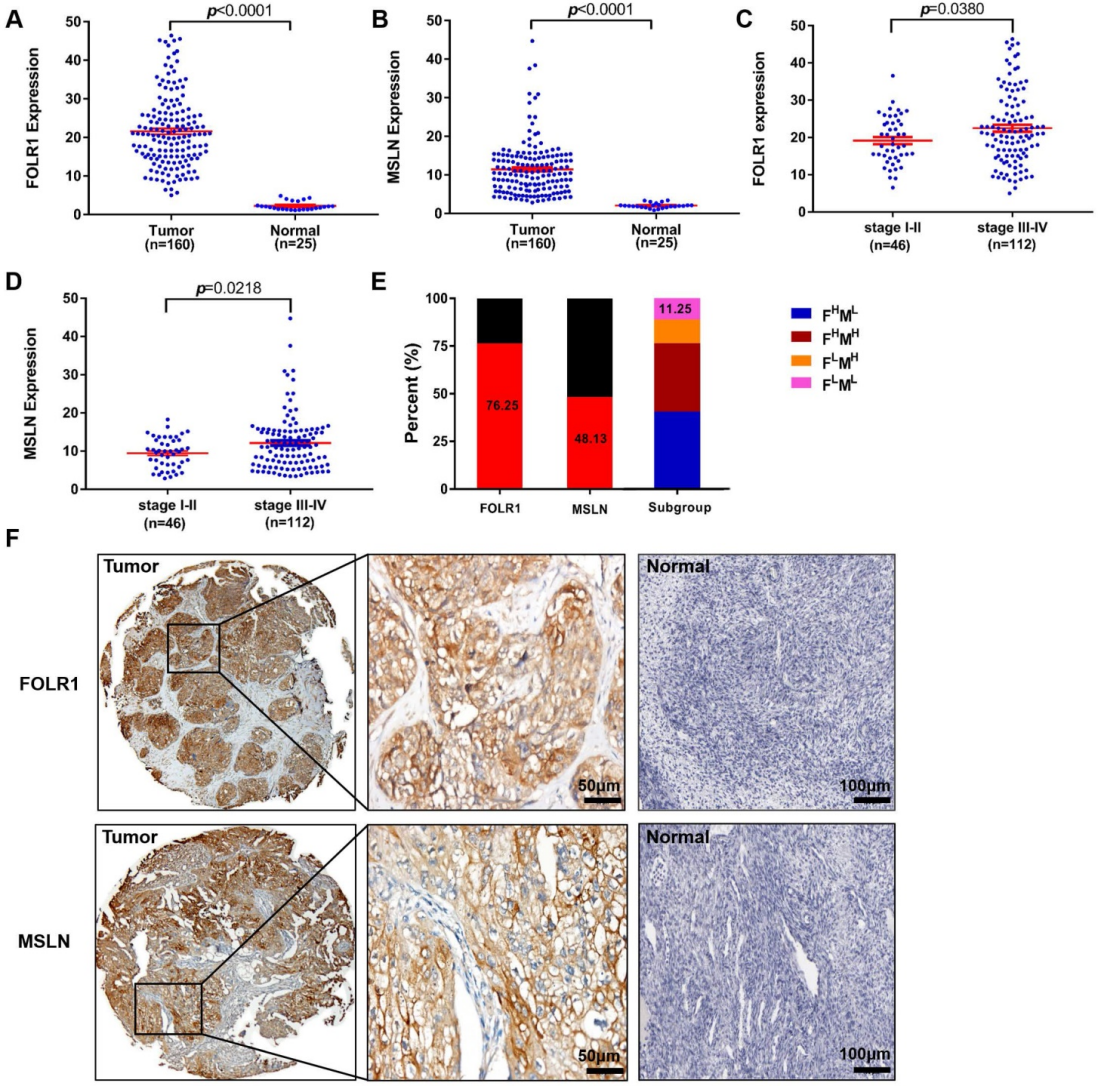
** Upregulated FOLR1/MSLN expression in OV tissues was associated with different stages of disease in patients.** (A, B) The expression of FOLR1/MSLN in OV tissues was significantly higher than that in normal tissues. (C, D) The expression of FOLR1/MSLN in OV tissues was significantly higher in advanced stages of the disease. (E) The proportion of FOLR1/MSLN-positive patients in the clinical cohort. Subgroup analysis showed that the percentages of FOLR1^high^/MSLN^low^, FOLR1^high^/MSLN^ high^, FOLR1^low^/MSLN^high^, and FOLR1^low^/MSLN^low^ patients were 40.63% (65/160), 35.62% (57/160), 12.50% (20/160), and 11.25% (18/160), respectively. (F) Representative photomicrographs of FOLR1 and MSLN expression in ovarian carcinoma tissue and the normal ovary control group. The scale bar represents 50 µm in tumors and 100 µm in normal controls.

Figure 2 displays the results for IHC staining. FOLR1 and MSLN were highly and specifically stained on the surface of ovarian tumor cells, while almost no significant staining was observed for FOLR1 or MSLN in normal ovarian tissue specimens (Figure 2F). FOLR1 and MSLN were highly expressed in OV tissues (n=160) compared with normal tissues (n=25) (*p* <0.0001, Figure 2A; *p* <0.0001, Figure 2B). Positive staining for FOLR1 or MSLN in the present study was observed in 122/160 patients (76.25%) and 77/160 patients (48.13%), respectively (Figure 2E). Only 11.25% of samples were negative for both proteins (Figure 2E). The expression of FOLR1 and MSLN was significantly associated with the FIGO stage, and late-stage disease (stage III-IV) had higher expression than the early-stage disease (stage I-II) (*p* =0.0380, Figure 2C; *p* =0.0218, Figure 2D; Table S3).

### Generation and proliferation of Tandem CAR-T cells

**Figure 3:**
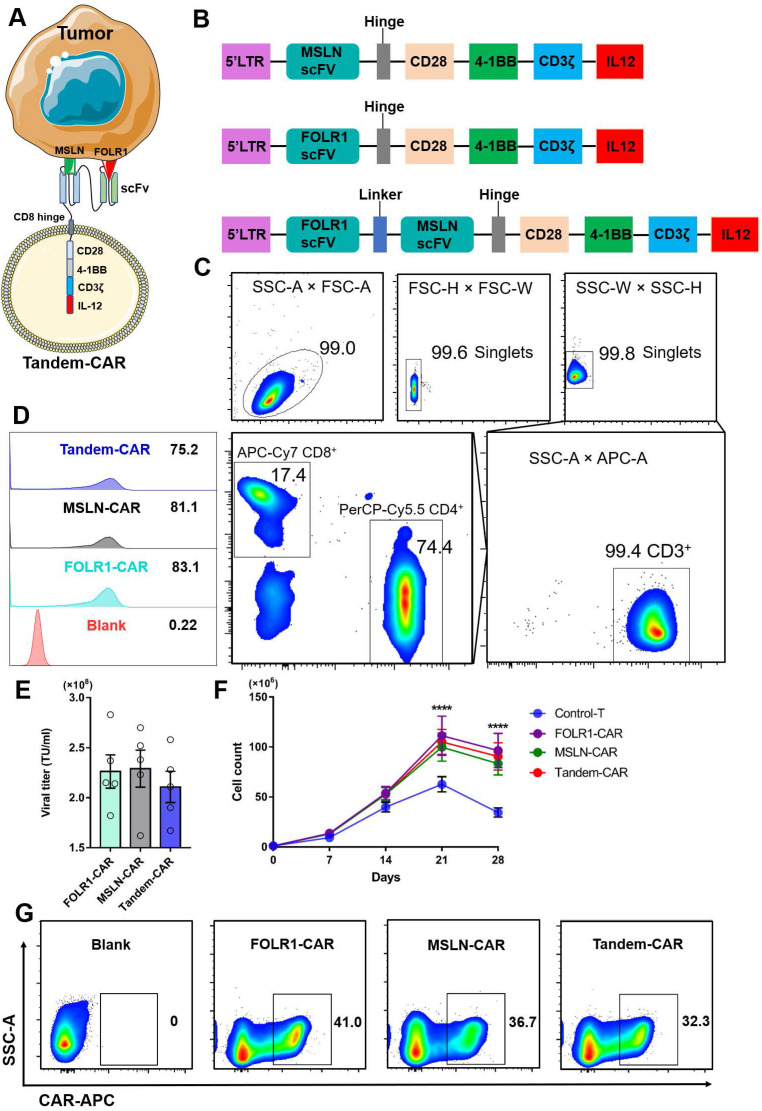
** Construction of Tandem-CAR.** (A) Schematic representation of Tandem CAR-T cells. (B) The structure of lentiviral vectors encoding various CARs used to construct modified T cells. (C) Sorting and characteristics of CD3^+^ T cells isolated from PBMCs. (D, E) The titers of the three lentiviruses were determined by flow cytometry. (F) Cell counts of the different CAR-T cells upon stimulated by SNU119 cells (FOLR1^+^MSLN^+^) at 21 and 28 days (n = 3). Data are shown as the mean ± SD (****P < 0.0001). (G) The expression levels of CARs were confirmed by flow cytometry.

The schematic of a Tandem-CAR is shown in Figure 3A, and three lentiviral vectors encoding various CARs (anti-MSLN, anti-FOLR1, and anti-FOLR1-MSLN scFvs) are schematically represented in Figure 3B. CD3^+^ T cells were isolated from PBMCs by magnetic bead separation, and the proportions of CD3^+^, CD4^+^, and CD8^+^ T cells were 99.4%, 74.4% and 17.4%, respectively (Figure 3C). To improve the expression of CAR genes, we tested the infectivity of the corresponding concentrated virus by flow cytometry (FC, Figure 3D) and calculated the titers of the three viruses. The results showed that the titers of the three lentiviral vectors were all above 10^8^ TU/ml (Figure 3E). The transduction efficiencies of FOLR1-CAR, MSLN-CAR, and Tandem-CAR were 41.0%, 36.7%, and 32.3%, respectively (Figure 3G). A long-term proliferation experiment showed that the three types of CAR-T cells exhibited significantly higher proliferation than Control-T cells when interacting with SNU119 cells (FOLR1^+^MSLN^+^) at 21 and 28 days (*p* <0.0001, Figure 3F), and this enhanced proliferation was antigen-dependent since it was not observed in the Control-T group (Figure 3F).

### Selection of target cells and cytotoxicity of CAR-T cells* in vitro*

**Figure 4:**
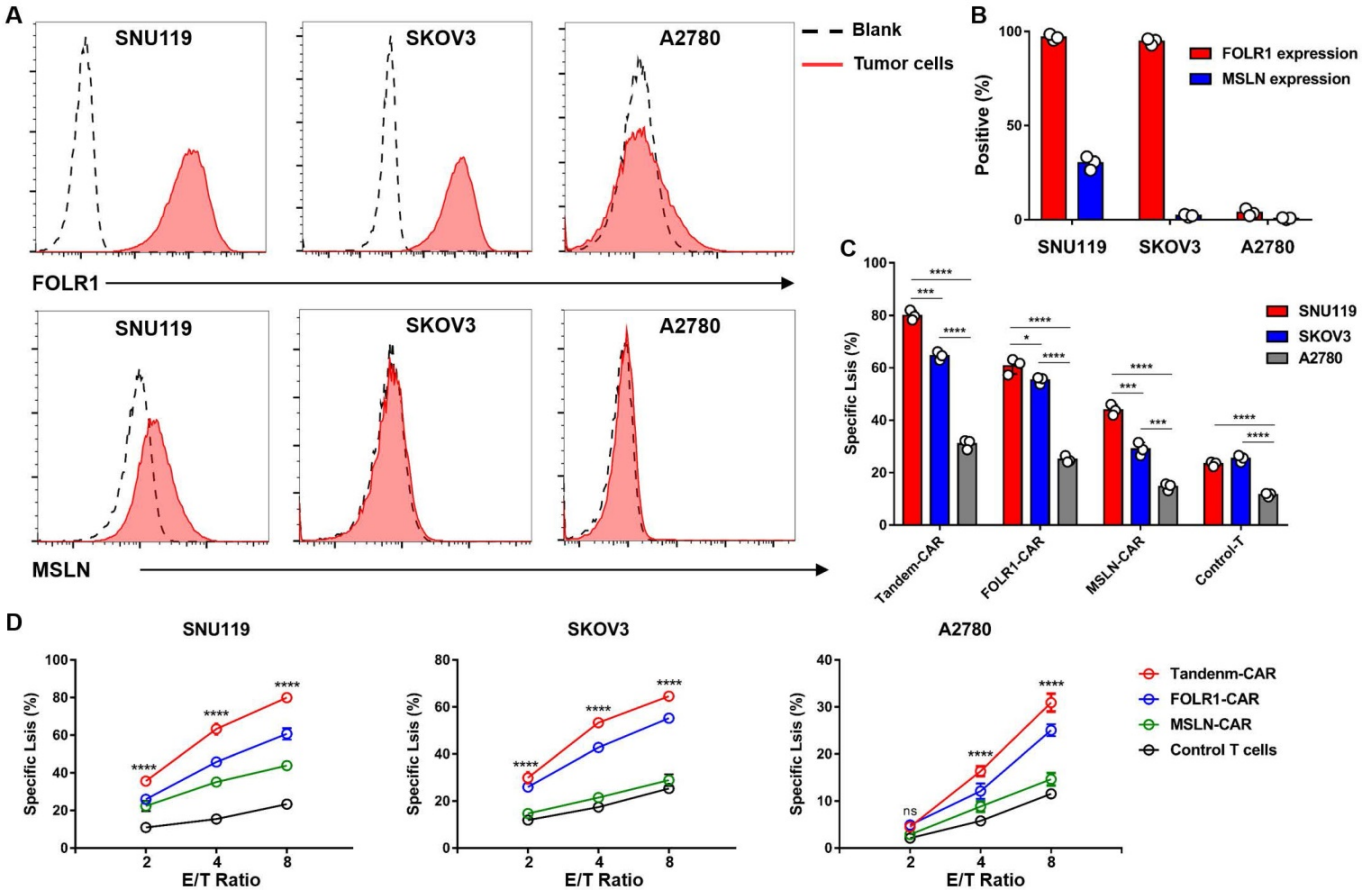
** Tandem CAR-T cells were activated and lysed antigen-positive target cells.** (A) The expression levels of FOLR1 and MSLN in OV cell lines were detected by FC. (B) The expression levels of the two antigens in tumor cells are shown in the histogram. (C) Levels of specific tumor cell lysis by CAR-T cells against three cancer cell lines after incubation at an E:T ratio of 8:1 for 18 hours. (D) CAR-T cells cocultured with SNU119, SKOV3, or A2780 cells at different E:T ratios for 18 hours. Supernatants were collected to detect cytotoxic activity. All data are shown as the mean ± SD (n = 3). *P < 0.05, **P < 0.01, ***P < 0.001, and ****P < 0.0001.

FC results indicated that the OV cell line SNU119 is an ideal target cell due to its high expression levels of both MSLN and FOLR1 (Figure 4A, B). SKOV3 cells expressed FOLR1 but lacked MSLN expression, and A2780 cells did not express FOLR1 or MSLN (Figure 4A, B). After transduction and two weeks of *in vitro* expansion, the cytotoxicity of CAR-T cells was tested by an LDH release assay at varying E:T ratios. As expected, Tandem-CAR-T cells exhibited stronger antitumor activity than single-target CAR-T cells at different E:T ratios in a dose- and antigen-dependent way (Figure 4D). Both Tandem-CAR T cells and FOLR1-CAR T cells could efficiently lyse FOLR1-positive cells (SNU119 and SKOV3) but not FOLR1-negative cells at the low ratio of 2:1 (*p* <0.0001, Figure 4D), whereas MSLN-CAR and Control-T cells showed lower and equivalent activity in lysing any of the MSLN-negative target cell lines tested (SKOV3 and A2780, Figure 4D). When cocultured with SNU119^MSLN^ cells, MSLN-CAR T cells showed stronger tumor lysis than when cocultured with MSLN-negative cells (E:T=8,* p* <0.001, Figure 4C). The cytotoxicity of Tandem-CAR-T cells was higher than that of FOLR1-CAR and MSLN-CAR T cells at a ratio of 8:1 (n=3; 79.96% *vs* 60.69% and 43.87% against SNU119, *p* <0.0001; 64.54% and 55.25% *vs* 28.93% against SKOV3, *p* <0.0001, Figure 4D). Notably, when the E:T ratio was more than 2, Tandem-CAR and FOLR1-CAR T cells still had a significant killing effect on antigen-negative cells (A2780) compared with Control-T cells (*p* <0.0001, Figure 4D).

### Phenotype and activation of CAR-T cells* in vitro*

To evaluate the immunocompetence of CAR-T cells, T cell markers and the differentiation markers CD45RO and CD62L were measured after coculture with SNU119 cells for 48 hours. FC showed similar amounts of CD4^+^, CD8^+^, CD62L^+^, and CD45RO^+^ cells among all CAR-T cells except FOLR1-CAR T cells, which had lower percentages of CD8^+^ and central memory T (Tcm) cell subsets than did Tandem-CAR T cells (n=3; 54.70% *vs* 53.07%, CD8^+^, *p* =0.04; 11.67% *vs* 11.03%, Tcm, *p* =0.02; Figure S1C). The three types of CAR-T cell populations contained higher percentages of CD8^+^, central memory T cells (Tcm), and effector T cells (Tem) cells and a lower percentage of CD4^+^ cells than Control-T cells (*p* <0.0001, Figure S1C). The mean levels of degranulation marker perforin and granzyme B were upregulated in all three CAR-T cells and Tandem-CAR T cells showed the highest amount than that in Control-T cells (n=3; 36.07% *vs* 5.33%, granzyme B, *p* <0.0001; 34.0% *vs* 5.77%, perforin, *p* <0.0001, Figure S2C). Collectively, these results indicated that these CAR-T cells had intrinsic target-dependent cytotoxic activity and cytotoxicity of Tandem-CAR T cells on FOLR1- or MSLN-positive cancer cells was greater than single-target CAR-T cells *in vitro*.

### Cytokine production of CAR-T cells *in vitro*

**Figure 5:**
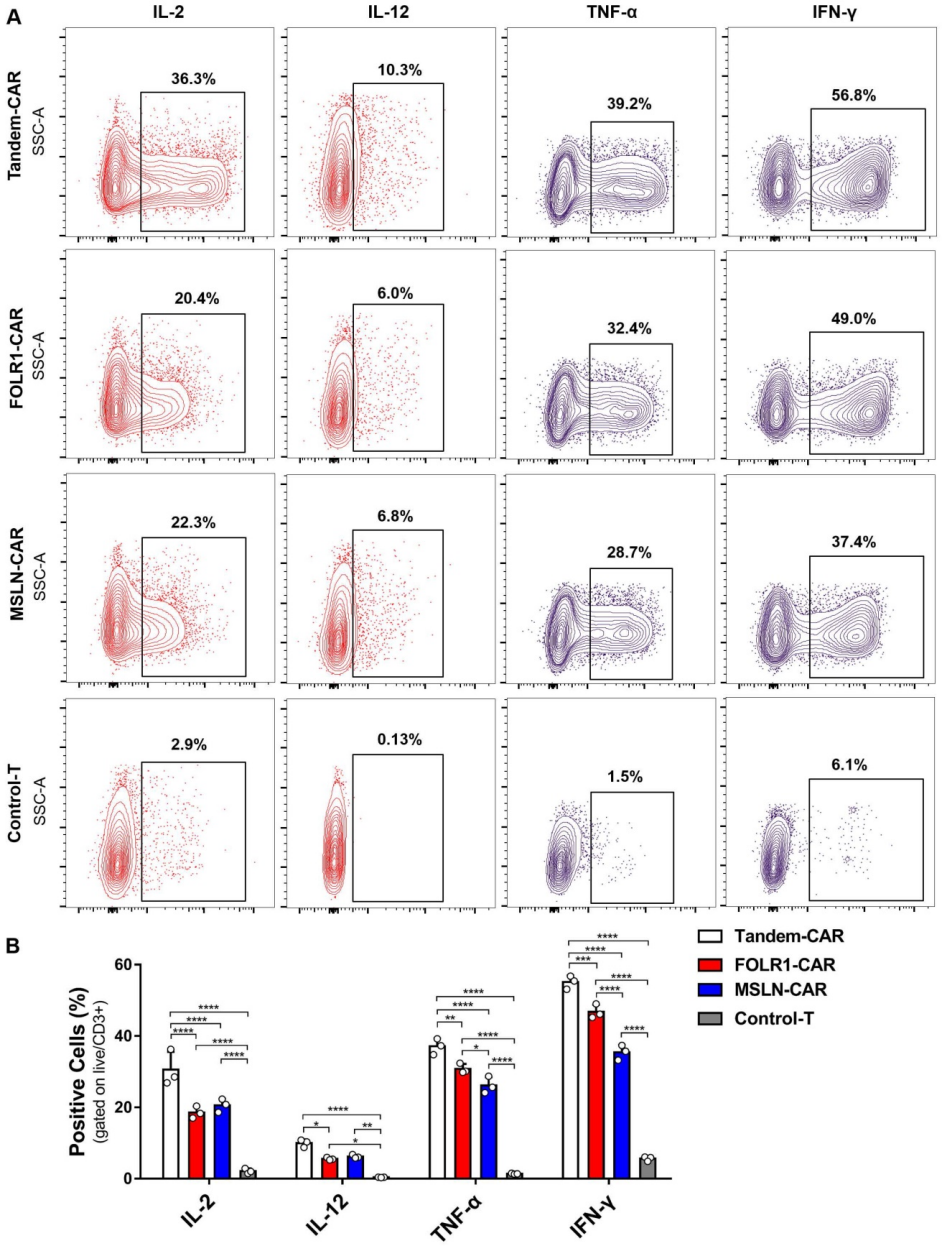
**Cytokine expression profiling of CAR-T Cell.** (A) CAR-T cells cocultured with SNU119 cells in triplicate at an E:T ratio of 2:1 for 24 h. The percentage of positive cells was measured by flow cytometric analyses. (B) Statistical analysis of cytokines. All data are shown as the mean ± SD (n = 3). *P < 0.05, **P < 0.01, ***P < 0.001, and ****P < 0.0001.

As shown in Figure 5B, the levels of various cytokines, including IL-2, IL-12, IFN-γ, and TNF-α, produced by Tandem-CAR and single-target CAR-T cells were significantly higher than those produced by Control-T cells when cocultured with SNU119 cells. As expected, all CAR-T cells secreted IL-12, whereas no secretion by Control-T cells was observed (Figure 5A). Tandem CAR-T cells produced higher levels of IL-2, IL-12, IFN-γ, and TNF-α than single-target CAR-T cells (Figure 5B). Interestingly, the cytokine release levels of MSLN-CAR T cells were slightly higher than those of FOLR1-CAR T cells, but the differences were not statistically significant (IL-2, IL-12, Figure 5B).

### Tandem CAR-T cells exhibited superior antitumor activity *in vivo*

**Figure 6:**
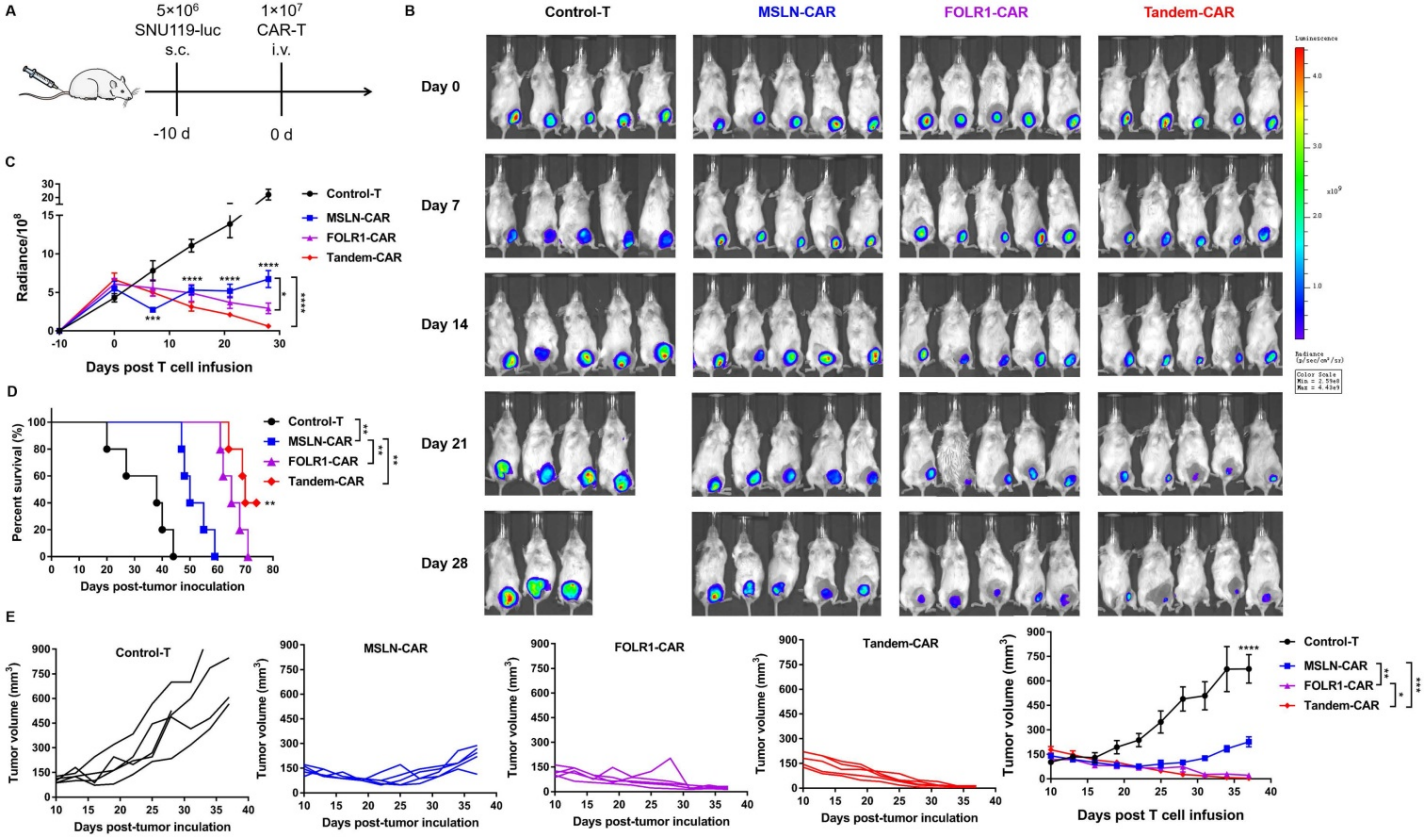
** Tandem CAR-T cells mediate a potent and lasting antitumor activity in xenografts.** The treatment scheme is shown in (A). B-NDG mice were injected subcutaneously (*s.c.*) with 5 ×10^6^ SNU119-Luc cells on day -10 and intravenously (*i.v.*) treated with 1 ×10^7^ Control, MSLN-CAR, FOLR1-CAR, or Tandem CAR-T cells on day 0. (B) BLI of mouse tumor burdens at the indicated time points, representative of all experiments (n = 5 mice per group). (C) Mouse tumor burdens (average radiance) are presented. (D) Survival analyses comparing mice treated with Tandem CAR-T cells with those treated with Control, MSLN-CAR, or FOLR1-CAR T cells. *P* values were calculated with a one-sided log-rank Mantel-Cox test. (E) Tumor volume was analyzed with calipers. Data are shown as the mean ± SD (n=5). *P < 0.05, **P < 0.01, ***P < 0.001, and ****P < 0.0001.

To further compare the antitumor efficacy of Tandem CAR-T cells and single-target CAR-T cells *in vivo*, tumor xenografts formed by SNU119 cells were established in B-NDG mice (n= 5). Mice were injected intravenously with various effector cells at 10 days after tumor engraftment (Figure 6A). We evaluated tumor progression by *in vivo* live imaging (Figure 6B) and caliper measurements (Figure 6E). Clear evidence showed that tumors progressed rapidly in the Control-T group, and two mice died due to a large tumor burden on days 20 and 28 (Figure 6B). The average tumor volume in the Control-T group increased to 711.2 mm^3^, whereas those in the MSLN-CAR, FOLR1-CAR, and Tandem CAR-T groups were 227.1 mm^3^, 21.7 mm^3^, and 5.0 mm^3^, respectively (*p* <0.0001, Figure 6E). Compared with Tandem-CAR and FOLR1-CAR T cells, MSLN-CAR T cells showed lower antitumor activity and only achieved a transient reduction in the tumor burden (*p* <0.001, *p* <0.01, Figure 6E), which was consistent with the total fluorescence intensity data (*p* <0.0001, *p* <0.05, Figure 6C). Notably, the antitumor activity of Tandem CAR-T cells exceeded that of FOLR1-CAR T cells (*p* <0.05, Figure 6E), and the tumor in one mouse was completely eradicated at day 28; the tumor did not recur through the end of the experiment (Figure 6B, D). In addition, treatment with Tandem CAR-T cells substantially prolonged treated mouse survival compared with single-target CAR-T cell treatment (*p* <0.01, Figure 6D). These results suggested that Tandem CAR-T cells displayed a superior antitumor activity over single-target CAR-T cells *in vivo*.

### Persistence and infiltration of CAR-T cells *in vivo*

We detected the number of human CD3^+^ T cells in treated mice at the experimental endpoint, and higher levels of circulating T cells were observed in the Tandem-CAR and FOLR1-CAR groups than in the Control-T and MSLN-CAR groups (*p* <0.0001, Figure S3). Using an anti-CD3ε mAb, anti-FOLR1 mAb, and anti-MSLN mAb, we examined the distribution of T cells and expression of FOLR1 and MSLN in tumor samples from mice by IHC analysis. As shown in Figure S4, the number of infiltrating CD3^+^ T cells in the three CAR-T cell treatment groups was significantly higher than that in the Control-T group (*p* <0.05, Figure S4). The infiltration rate of the Tandem-CAR T cell group was higher than that of the MSLN-CAR T and Control-T groups, while there was no significant difference compared with that of the FOLR1-CAR group (Figure S4), which was consistent with the results of Figure S3. For the expression of antigens in tumor tissues, the results indicated that the percentage of FOLR1^+^MSLN^+^ cancer cells was significantly decreased in the Tandem-CAR T cell group, while single-target CAR T cells only reduced the corresponding antigen level (*p* <0.0001, Figure S4). Combined with the results of Figure 6, these data indicate that it is conceivable that Tandem CAR-T cells have optimal antitumor activity *in vivo.*

## Discussion

CAR-T cell therapy, an innovative method with an 80-90% complete remission rate, has achieved unprecedented clinical efficacy in the treatment of B-cell malignancies 22, 23. Despite encouraging achievements, achieving efficacy with CAR-T cells in solid tumors is still elusive 11. In ovarian cancer, currently, multiple TAAs, such as MUC16, EpCAM, CD47, NY-ESO-1, HER2, MSLN and FOLR1, have been utilized in CAR-T cell-based immunotherapy, but these approaches have failed to replicate the success of CD19 CAR-T cells in hematopoietic cancers. The factors that contribute to the poor efficacy can be summarized as three major parts: lack of an ideal surface antigen, insufficient infiltration, and exhaustion of CAR-T cells in solid tumors. Given the above reasons, we developed a different strategy, using CAR-T cells that simultaneously target two antigens with secretion of IL-12 to reduce the likelihood of tumor antigen escape and potentially increase the infiltration and antitumor activity of CAR-T cells.

Here, we found that Tandem CAR-T cells could specifically recognize either FOLR1- or MSLN-positive tumor cells and exhibited a superior antitumor effect with a lower E:T ratio than single-target CAR-T cells. When engaged with SNU119 cells (FOLR1^+^MSLN^+^), the cytokine secretion of Tandem-CAR T cells was better than that of single-target CAR-T cells and Control-T cells. Compared with Control-T cells, all three types of CAR-T cells successfully expressed IL-12, and increased secretion of IL-12 can activate the functions of cytotoxic T cells, natural killer T cells, and NK cells, which is essential for enhancing the antitumor response 24. Furthermore, *in vivo* experiments also indicated that Tandem CAR-T cells could not only induce dual-antigen coverage but also elicit superior and persistent antitumor activity by increasing the infiltration of T cells into tumor tissue. Our findings add to the growing but scarce evidence concerning the application of the Tandem-CAR strategy in solid tumors, establishing a foundation for future clinical utility.

The safety and effectiveness of CAR-T cells have always been the focus of all research. In the present study, two antigens screened by bioinformatics and analysis of clinical specimens were almost undetectable in normal ovarian tissue, which met the requirement for safety. In terms of efficacy, the theoretical clearance rate of Tandem CAR-T cells against cancer cells was 88.75%, which was higher than that of the other two types of single-target CAR-T cells (Figure 2). As expected, our findings supported this hypothesis. When the E:T ratio was 8:1 and the target cell line was SNU119 (F^H^M^H^), we observed that the most significant killing efficiency was nearly 80% in the Tandem-CAR group, while none of the three types of CAR-T cells showed a high killing efficiency when exposed to antigen-negative cells (Figure 4). For SKOV3 cells (F^H^M^L^), the tumor lysis efficiency of the Tandem-CAR and FOLR1-CAR groups was significantly higher than that of the MSLN-CAR and Control-T groups, and there was no significant difference between the killing ability of MSLN-CAR T cells and that of Control-T cells. All these results indicated that the three types of CAR-T cells could specifically recognize and lyse antigen-expressing OV cells, which were also confirmed by the IHC results for mouse tumor tissue (Figure S4). Notably, compared with the controls, Tandem CAR-T cells still had a significant killing effect when the E:T ratio was 8:1 and A2780 cells were used as target cells, suggesting that Tandem CAR-T cells have a certain lytic activity against tumor cells with low antigen expression. The reasons behind this phenomenon are unknown, but two factors may contribute to the discrepancy. One possibility is that the detection limit of FC is high, and some tumor cells with low antigen levels are not detected but can be recognized by CAR-T cells. Recently, Siu *et al* demonstrated that myeloma cells expressing CD19 at very low levels could not be identified by FC; however, this expression level was sufficient to induce elimination by CD19 CAR-T cells 25. In addition, the secretion of IL-12 by CAR-T cells could activate T cell proliferation and augment antitumor effects 24, 26. However, further studies are needed to verify the specific mechanism.

Although FOLR1 and MSLN have been targeted in some CAR-T cell therapies, the antitumor effects exhibited in these studies have been modest. For example, two studies have indicated that MSLN-CAR T cells can only induce slight regression of cervical cancer 27 and malignant pleural mesothelioma 28. Other studies have shown modest antitumor response by FOLR1-CAR T cells in xenograft models of gastric cancer 29 and triple-negative breast cancer 30. Clearly, such moderate antitumor effects would not meet the clinical requirements for improving efficacy. Of note, all CARs in the above studies employed one costimulatory molecule, such as CD28 or 4-1BB, while our studies used third-generation CARs since it has been reported that third-generation CARs exert more potent antitumor activity than second-generation CARs, despite some controversial views 9, 31, 32. Here, we provide the first evidence that Tandem CAR-T cells targeting FOLR1 and MSLN had superior antitumor performance compared with single-target CAR-T cells *in vivo* and had the potential to eradicate OV tumors (Figure 6).

IL-12, a proinflammatory cytokine with potent tumor-suppressive activity, exhibits some immunostimulatory functions, such as improving antigen presentation and mitigating antigen-negative escape 15, 16. In terms of T cell stimulation and costimulation, IL-12 has been shown to act as signal 3, inducing the proliferation and activation of T cells 33, 34. Furthermore, a recent study showed that intratumoral IL-12 delivery could not only reshape the immunosuppressive tumor microenvironment (TME) but also boost the cytotoxicity of CAR-T cells in a glioblastoma model 24. Given these properties of IL-12, we speculated that modifying CAR-T cells to express IL-12 might enhance the antitumor response in ovarian cancer. We found that all three types of CAR-T cells successfully secreted IL-12, significantly increased the proliferation of T cells and prolonged the survival of tumor-bearing mice compared with the Control-T cells (Figure 3, 6). The levels of other cytokines secreted by CAR-T cells, such as IL-2, TNF-α, and IFN-γ, were also significantly higher than those secreted by Control-T cells, and the most obvious cytokine secretion capacity was observed in the Tandem-CAR group.

Using an ectopic xenograft model with high expression of FOLR1 and MSLN, complete tumor eradication was achieved in one of five treated mice in the Tandem-CAR group, with tumor outgrowth observed in the single-target CAR-T groups. In addition, we found that MSLN-CAR T cells maintained transient antitumor activity but failed to achieve tumor control, possibly due to the limited antigen expression in SNU119 cells and the low percentage of CD3^+^ cells in the peripheral circulation (Figure S3). Reportedly, the infiltration and persistence of T cells in tumor tissues are closely related to tumor suppression. Our results showed that the number of CD3^+^ cells in mouse peripheral blood was significantly increased in the Tandem-CAR and FOLR1-CAR groups compared with the MSLN-CAR and control groups; this phenomenon was also observed in the IHC data (Figure S4). Altogether, these data demonstrated that the transferred Tandem CAR-T cells survived *in vivo* and trafficked into antigen-positive ovarian cancer tissue, mitigating antigen escape and significantly increasing tumor cell-killing activity compared with single-target CAR-T cells.

In summary, this study provides experimental evidence that Tandem CAR-T cells capable of secreting IL-12 have an effective and durable antitumor effect; this approach can be a feasible strategy for adoptive T cell therapy and shows promising application potential for other solid tumors.

## Supplementary Material

Supplementary figures and tables.

## Abbreviations

CAR-T: chimeric antigen receptor T cells
FC: flow cytometry
FOLR1: folate receptor 1
IHC: immunohistochemistry staining
IFN-γ: interferon-gamma
IL-2: interleukin-2
IL-12: interleukin-12
MSLN: mesothelin
OV: Ovarian cancer
TAAs: tumor-associated antigens
Tcm: central memory T cells
Tem: effector T cells
TNF-α: tumor necrosis factor-α
