# Supplementary material for: Tandem CAR-T cells targeting FOLR1 and MSLN enhance the antitumor effects in ovarian cancer
Source: Int J Biol Sci. 2021 Oct 22;17(15):4365–76. doi: 10.7150/ijbs.63181 (PMC8579462; doi:10.7150/ijbs.63181)
Supplement: Supplementary file 1 — Supplementary figures and tables. [file ijbsv17p4365s1.pdf]

Supplemental Materials for

Tandem CAR-T cells targeting FOLR1 and MSLN enhance the antitumor effects in ovarian cancer.

Zhen Liang[1, #], Jiao Dong[1, 2, #], Neng Yang[1], Si-Di Li[1], Ze-Yu Yang[3], Rui Huang[4], Feng-Jie Li[1], Wen-Ting Wang[1], Jia-Kui Ren[1], Jie Lei[5], Chen Xu[6], Dan Wang[1], Yan-Zhou Wang[1], Zhi-Qing Liang[1, *]

[1] Department of Obstetrics and Gynecology, Southwest Hospital, Army Medical University (Third Military Medical University), Chongqing, China.

[2] Department of Obstetrics and Gynecology, Guangyuan Traditional Chinese Medicine Hospital, Guangyuan, China.

[3] Breast and Thyroid Surgical Department, Chongqing General Hospital, University of Chinese Academy of Sciences, Chongqing, China.

[4] Institute of Pathology and Southwest Cancer Center, Southwest Hospital, Army Medical University (Third Military Medical University), Chongqing, China.

[5] Department of Internal Medicine, Hui Long-Ba Town Hospital, Chongqing, China.

[6] Department of Hepatobiliary Surgery, Xijing Hospital, The Fourth Military Medical University, Xi'an, China.

# These authors contributed equally to the work.

Declarations of interest: none

***Corresponding author:**

Dr. Zhi-Qing Liang, Ph.D.

Professor

Department of Obstetrics and Gynecology,

Southwest Hospital,
Army Medical University (Third Military Medical University),
30 Gaotanyan Main Street, Shapingba, Chongqing, China 400038
Phone: +86-23-68765401
ORCID: 0000-0001-6459-2541
E-mail: lzq@tmmu.edu.cn, zhilzliang@outlook.com, or zhilzliang@163.com

This file includes the following:

Methods

Supplemental Tables 1 to 5

Supplemental Figures 1 to 4

Methods

Identification of upregulated genes from the GEO database

We processed the downloaded files with RStudio software (3.5.3 version, Vienna, Austria) and then standardized, calibrated, and log 2 converted the resulting data. The cutoff criteria for upregulated gene screening were $p< 0.01$ and $|\log FC|\geq 2$. The coexpressed upregulated genes in these four gene expression profiles were evaluated by Venn diagram analysis. After the coexpressed upregulated genes were identified, cluster analysis was performed by RStudio software with a heatmap package (heatmap) to further reveal significant differences between the normal and OV tissues. The volcano plot of these four gene expression profiles was generated with R software, and the criteria $p< 0.01$ and $|\log FC|\geq 2$ were used.

Immunohistochemistry (IHC) staining and quantification

In brief, tissue slides were heated and deparaffinized in xylene immediately. After rehydration in a graded series of ethanol solutions, tissue slides were submerged in citrate antigen retrieval solution (pH=6.0) and heated in a microwave oven for antigen retrieval, and the activity of endogenous peroxidases was blocked by hydrogen peroxide. Subsequently, the sections were incubated with an anti-FOLR1 rabbit polyclonal antibody (Abcam, 1:100 dilution) and anti-MSLN mouse monoclonal antibody (Invitrogen, 1:10 dilution) overnight at 4°C. Negative IHC staining controls obtained by omitting the primary antibodies were routinely used. After washing, the slides were incubated with corresponding secondary antibodies (ZSGB-BIO, China), stained with diaminobenzidine (DAB; DAKO, S196130-2,

Denmark). Finally, the samples were counterstained with hematoxylin and dehydrated.

The IHC staining was scored from 0 to 3 (representing negative, weak, moderate, and strong staining, respectively). Based on the percentage of positive tumor cells observed per tissue, the extent of IHC staining was scored: 1 (0-25%), 2 (26-50%), 3 (51-75%), and 4 (76-100%). The final staining index (0-12) of each sample was obtained from the multiplying results of two scores. High and low expression was defined as a final score of 6-12 and 0-4, respectively. Alternatively, using integrated optical density (IOD) analyzed with Image-Pro® Plus software (version 6.0; Media Cybernetics, Inc.) to show the quantitative analysis results.

Cells lines

The HEK293T cell lines and human OV cell lines SNU119, SKOV3, and A2780 were obtained from the Type Culture Collection of the Chinese Academy of Sciences (Shanghai, China). These cells were cultured in RPMI-1640 medium or DMEM supplemented with 10% fetal bovine serum (FBS, HyClone or Gibco), 100 μg/ml penicillin, and 100 U/ml streptomycin (Invitrogen). SNU119, SKOV3, and A2780 target cells were transduced with a lentiviral vector encoding the firefly luciferase gene to generate SNU119-Luc, SKOV-3-Luc, and A2780-Luc cells.

Lentiviral vector production and CAR-T Cell production

Plasmids were cotransfected with the packaging plasmids psPAX2 and pMD2.G

(ratio 2.5:2:1) into HEK293T cells. Supernatants containing viral particles were collected at 24 and 48 hours after transfection, pooled, filtered, concentrated by a Lenti-X concentrator (Clontech), resuspended in PBS supplemented with 1% FBS, aliquoted, and used immediately or stored at -80°C. Viral titers were measured by flow cytometry (FC) with serial dilutions.

CD3$^+$ T cells were isolated from peripheral blood mononuclear cells (PBMCs, StemCell Technologies) using an EasySep human T cell isolation kit (StemCell Technologies) and cultured in RPMI 1640 medium supplemented with 10% FBS (Gibco), 0.1 mg/mL streptomycin (Gibco), 100 U/mL penicillin (Gibco), 2 mM L-glutamine (Gibco), and 100 IU/ml recombinant human IL-2 (Miltenyi Biotec). T cells were activated by Human CD3/CD28 T Cell Activator (Stem Cell Technologies) for 24 hours and infected with lentiviruses containing the different CAR transgenes in RetroNectin-coated (Takara, 32 μg/mL) 24-well plates at a multiplicity of infection (MOI) of 10. The T cells were spin-infected at 2,000 g for 2 hours with the virus at 32°C. After transduction, the cells were cultured in T cell medium containing 100 U of IL-2 per ml and expanded for approximately 10 days. Activated T cells were cultured at concentrations between 0.5 and 1 × 10^6 cells/mL.

Flow cytometry analysis

Up to 10^6 cells were stained with fluorochrome-conjugated antibodies for 30 min at 4°C in the dark. T cells were stained with an APC-conjugated anti-$F(ab')_2$ antibody (Jackson ImmunoResearch) to determine the expression of the desired CAR transgene.

FOLR1 and MSLN expression in tumor cell lines was detected with a PE-conjugated anti-FOLR1 antibody (BioLegend) and an APC-conjugated anti-MSLN antibody (*R&D* Systems). T cell surface molecules were stained with anti-human CD3 (PerCP-Cy5.5, BD), CD4 (APC-Cy7, BioLegend), CD8 (FITC, BD), CD62L (PE, BioLegend), and CD45RO (APC, BD) antibodies. The phenotype of CAR-T cells was determined by evaluating the percentages of $CD62L^{+}CD45RO^{+}$ central memory T cells (Tcm) and $CD62L^{-}CD45RO^{+}$ effector T cells (Tem) after 48 h of coculture with SNU119 cells. The non-transduced T cells were treated equally and served as Control-T cells. Fluorescence was analyzed using a BD FACSCanto flow cytometer, and data were analyzed using FlowJo V10 software.

Cytokine Release and cell proliferation assays

Cytokine production was detected by intracellular cytokine staining of T cells. CAR-T cells were incubated with SNU119 cells at an effector-to-target (E:T) cell ratio of 2:1 in 200 μL of complete medium (without IL-2) and then incubated with Golgi Plug to block exocytosis of cytokines for 4 h (BD Biosciences). Following this, the cells were stained for surface markers (CD3/APC, CD4/APC-Cy7, and CD8/FITC), followed by fixation, permeabilization, and staining for intracellular proteins with the Cytofix/Cytoperm Fixation/Permeabilization Solution Kit (BD Biosciences). Intracellular cytokine staining was performed with PE/Cy7-conjugated anti-human IFN-γ, PE-conjugated anti-human TNF-α, PerCP-Cy5.5-conjugated anti-human IL-12, and BV421-conjugated anti-human IL-2 antibodies (BioLegend). For the proliferation

assay, T cells (1×10^6) were plated in 6-well plates (without IL-2) and stimulated weekly with SNU119 cells at an E:T ratio of 1:1. Cell numbers and size were evaluated using a Bio-Rad TC20TM cell counter (USA). For the degranulation assay, CAR-T cells were cocultured with SNU119 cells at an E:T ratio of 1:1 in complete medium containing the anti-human perforin-PerCP-Cy5.5 and anti-human granzyme B-Pacific Blue antibody (BioLegend) for 1 h, followed by incubation with a Golgi Plug (BD Biosciences) for 3 h.

Cytotoxicity assay

CAR-T cells were cocultured with human ovarian cell lines with different expression levels of FOLR1 and MSLN at different ratios for 18 hours to assess cytotoxicity and maintain a total cell concentration of 1×10^6/mL. The amount of lactate dehydrogenase (LDH) in the supernatant was quantified using a CytoTox 96® Non-Radioactive Cytotoxicity Assay kit (Promega). The percent specific killing of target cells (lytic activity) was calculated according to the manufacturer's instructions based on absorbance values (490 nm) subtracted from the culture medium background. Calculations for % cell lysis were consisted with previously described [1].

Xenograft mouse model

For *in vivo* study of CAR-T cell functions, 6- to 8-week-old female NOD-*Prkdc*^{scid}*IL2rg*^{tm1}/Bcgen (B-NDG, Biocytogen) mice were subcutaneously injected with 5×10^6 SNU119-luciferase cells in the left groin. Approximately 10 days later, the tumor volume reached approximately 50 to 100 mm^3. The tumor-bearing

mice were randomly assigned into 4 groups and were injected i.v. with 1×10^7 untransfected (Control-T), FOLR1-CAR, MSLN-CAR, or Tandem-CAR T cells in 200 μl of PBS. An IVIS imaging platform with Living Image software (PerkinElmer) was used to evaluate the bioluminescence imaging (BLI) and BLI data was given as the average flux (photons per second/area[mm^2]). Additionally, tumor volume was measured every three days and calculated based on the equation: length × $(width)^2$ × 0.5. Tumor BLI was performed until death or sacrifice when tissues were harvested for analysis. Mice were euthanized when maximum tumor diameters exceeded 2 cm or bodyweight loss over 20%. We collected peripheral blood samples when mice were sacrificed, red blood cells were lysed using AR1118 Lysing Buffer (Boster Biological Technology), and the remaining cells were stained with an APC-conjugated anti-CD3 antibody for FC. To analyze the intratumoral infiltration of T cells, IHC staining was performed using standard procedures. The expression of FOLR1 and MSLN in the different groups was also determined by IHC staining. Primary rabbit anti-human CD3ε (CST), rabbit anti-human FOLR1 (Abcam) and mouse anti-human MSLN antibodies (Invitrogen) and a biotinylated secondary goat anti-rabbit/mouse antibody were used.

Table S1. Characteristics of the GEO microarray datasets

Chip serial number	OV patients	Normal patients	Platform
GSE28721	8	2	GPL6884 Illumina HumanWG-6 v3.0 expression beadchip<br>GPL6947 Illumina HumanHT-12 V3.0 expression beadchip
GSE66957	57	12	GPL15048 Rosetta/Merck Human RSTA Custom Affymetrix 2.0 microarray
GSE36668	4	4	GPL570 [HG-U133_Plus_2] Affymetrix Human Genome U133 Plus 2.0 Array
GSE4122	32	14	GPL201 [HG-Focus] Affymetrix Human HG-Focus Target Array

Table S2. Descriptive statistics for clinic-pathological features in ovarian cancer patients

Variable	Number of patients	Percentage (%)
Age		
≤50	77	48.13
>50	83	51.87
Histological subtypes		
Serous	100	62.50
Mucinous	33	20.62
Endometrioid	17	10.63
Others[a]	10	6.25
Grade of differentiation		
1	19	12.03
2	35	22.15
3	104	65.82
FIGO stage		
I	9	5.70
II	37	23.42
III	79	50.00
IV	33	20.88
Lymph node metastasis		
Positive	42	26.58
Negative	116	73.42
Progression status		
No recurrence	31	19.62
Recurred	127	80.38

[a] Malignant Brenner tumor, 1 case; Clear-cell, 3 cases; Borderline mucinous tumor, 2 cases; Squamous cell carcinoma, 2 cases; Yolk sac tumor, 2 cases.

Table S3. Association between FOLR1 and MSLN expression and clinic-pathological criteria

Variable	FOLR1 expression (n=160)			MSLN expression (n=160)		
	High	Low	*P* value	High	Low	*P* value
Age			0.791			0.198
≤50	58 (36.25%)	19 (11.87%)		33 (5%)	44 (43.13%)	
>50	64 (40.00%)	19 (11.88%)		44 (11.25%)	39 (40.62%)	
Histological subtypes			0.178			0.048*
Serous	76 (47.50%)	24 (15.00%)		58 (13.75%)	42 (48.75%)	
Mucinous	22 (13.75%)	11 (6.88%)		9 (0.62%)	24 (20.00%)	
Endometrioid	16 (10.00%)	1 (0.62%)		8 (1.25%)	9 (9.38%)	
Others[a]	8 (5.00%)	2 (1.25%)		2 (0.62%)	8 (5.63%)	
Grade of differentiation			0.515			0.507
1-2	43 (27.22%)	11 (6.96%)		24 (3.17%)	30 (31.01%)	
3	78 (49.37%)	26 (16.45%)		52 (12.66%)	52 (53.16%)	
FIGO stage			0.048*			7.518E-05*
I-II	40 (25.32%)	6 (3.80%)		16 (0.0%)	30 (29.11%)	
III-IV	81 (51.26%)	31 (19.62%)		60 (15.82%)	52 (55.06%)	
Lymph node metastasis			0.178			0.008*
Positive	29 (18.35%)	13 (8.23%)		24 (7.59%)	18 (18.99%)	
Negative	92 (58.23%)	24 (15.19%)		52 (8.23%)	64 (65.19%)	
Progression status			0.285			0.062
No recurrence	26 (16.45%)	5 (3.17)		9 (0.63%)	22 (18.99%)	
Recurred	95 (60.13%)	32 (20.25%)		67 (15.19%)	60 (65.19%)	
Survival status			0.001*			0.007*
Living	66 (41.25%)	9 (5.63%)		32 (3.75%)	43 (43.12%)	
Dead	56 (35.00%)	29 (18.12%)		45 (12.50%)	40 (40.63%)	

[a] Malignant Brenner tumor, 1 case; Clear-cell, 3 cases; Borderline mucinous tumor, 2 cases; Squamous cell carcinoma, 2 cases; Yolk sac tumor, 2 cases.

Table S4. Univariate and multivariate analyses of different prognostic parameters

Variable	Case	Univariate analysis				Multivariate analysis			
						OS(M)		DFS(M)	
		OS(M)	*P*	DFS(M)	*P*	HR (95% CI)	*P*	HR (95% CI)	*P*
Age at Diagnosis			0.082		0.031*		0.343		0.460
≤50	77	62.91		50.14		1.0		1.0	
>50	83	54.58		36.72		1.251 (0.788-1.986)		0.864 (0.587-1.273)	
Histological subtypes			0.000*		0.000*		0.032*		0.078
Serous	100	51.09		36.45		1.0		1.0	
Non-serous[a]	60	71.08		54.40		0.553 (0.321-0.950)		0.700 (0.472-1.040)	
Grade			0.003*		0.005*		0.128		0.026*
1-2	54	64.43		49.06		1.0		1.0	
3	104	54.73		39.01		1.556 (0.880-2.749)		1.693 (1.065-2.693）	
FIGO stage			0.000*		0.000*		0.000*		0.000*
I-II	46	79.80		68.80		1.0		1.0	
III-IV	112	49.11		31.62		12.111 (4.128-35.536)		6.652 (3.620-12.224）	
Lymph-node metastasis			0.000*		0.000*		0.000*		0.001*
Negative	116	67.98		50.40		1.0		1.0	
Positive	42	30.60		20.48		3.245 (1.981-5.315)		2.042 (1.327-3.141）	
FOLR1 Expression			0.004*		0.0078*		0.047*		0.697
FOLR1 ^{low}	38	49.05		37.47		1.0		1.0	
FOLR1 ^{high}	122	61.56		44.96		0.564 (0.321-0.991)		0.918 (0.596-1.414）	
MSLN Expression			0.008*		0.0057*		0.505		0.353
MSLN ^{low}	134	60.39		45.09		1.0		1.0	
MSLN ^{high}	26	49.31		33.35		1.228 (0.672-2.246)		1.268 (0.769-2.091)	

[a] Mucinous, 33 cases; Endometrioid, 17 cases; Malignant Brenner tumor, 1 case; Clear-cell, 3 cases; Borderline mucinous tumor, 2 cases; Squamous cell carcinoma, 2 cases;

166	Yolk sac tumor, 2 cases.

Table S5. The relevance expression of FOLR1 and MSLN in ovarian cancer as judged by immunocytochemistry

MSLN expression (n=160)	FOLR1 expression (n=160)		
	Positive	Negative	Total
Positive	57 (35.63%)	20 (12.50%)	77
Negative	65 (40.63%)	57 (35.62%)	122

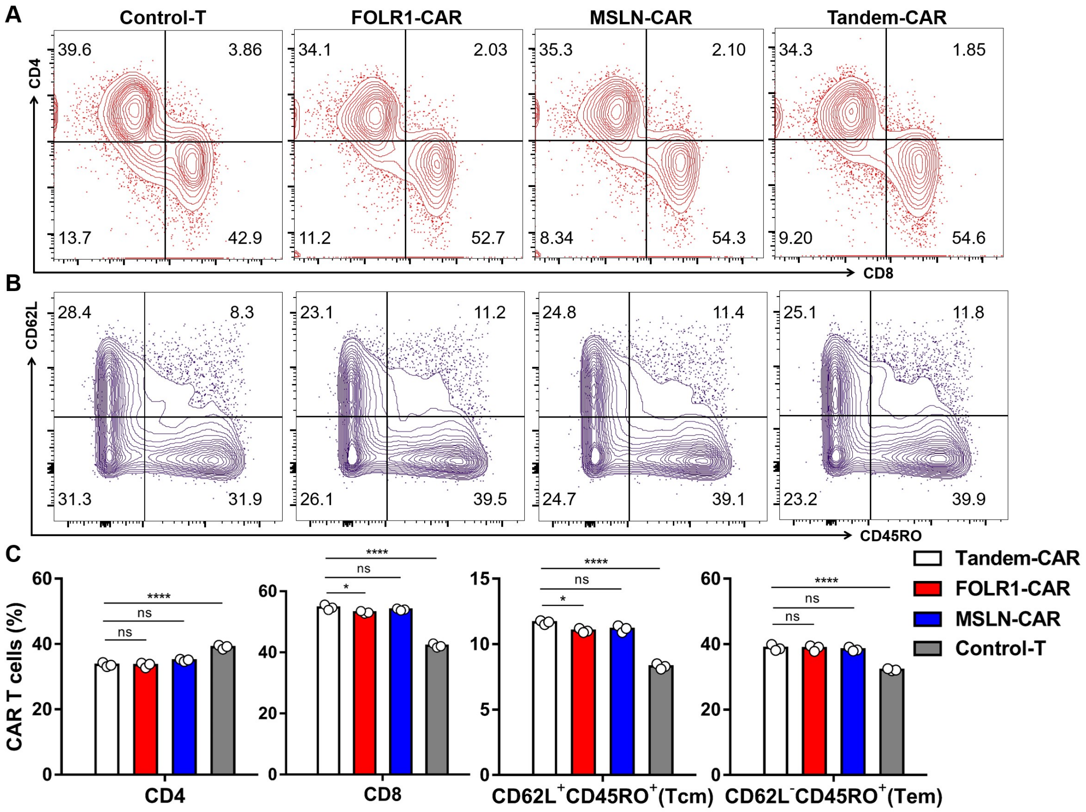

Figure S1. Phenotype of Tandem-CAR T cells compared to that of single-target CAR-T cells. **(A, B)** Flow cytometric analysis of cell-surface CD4, CD8, CD62L, and CD45RO phenotypic marker staining of Control-T cells and CAR-T cells after expansion. **(C)** The phenotype of CAR-T cells was determined by measuring the percentages of $CD62L^+CD45RO^+$ central memory T cells and $CD62L^-CD45RO^+$ effector T cells. Data are shown as the mean ± SD (n=3). ns: not significant, *$P < 0.05$, and ***$P < 0.001$.

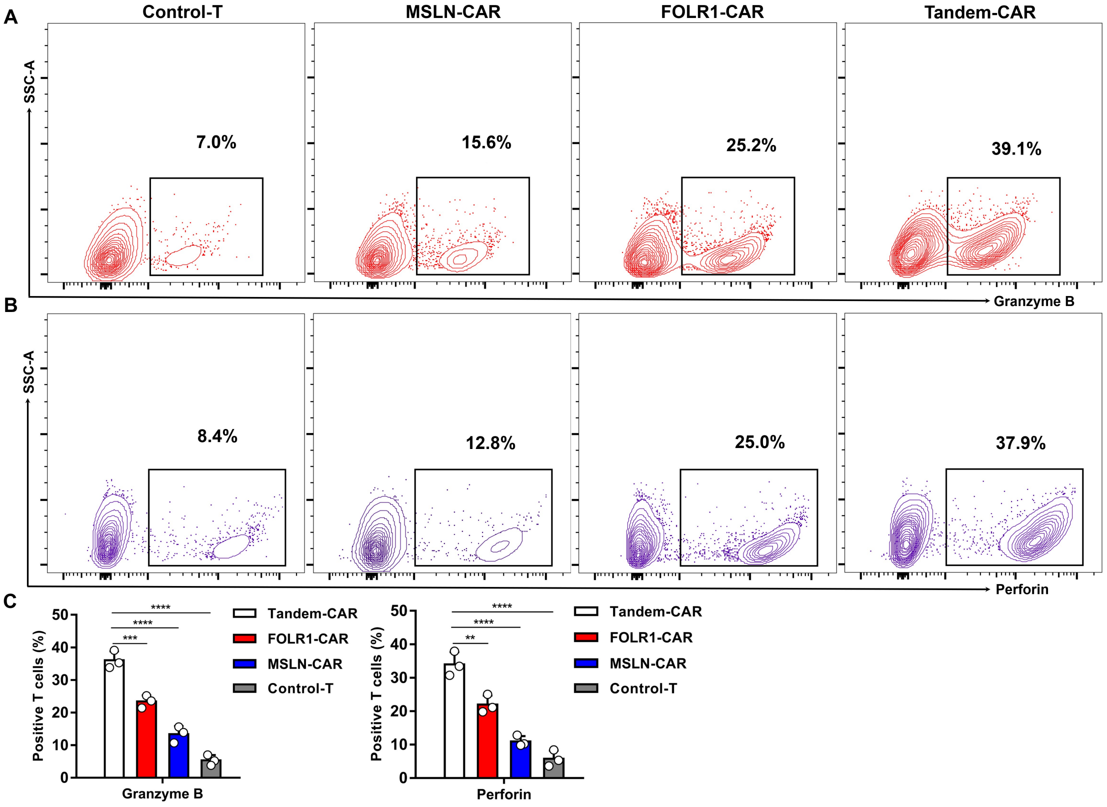

Figure S2. Degranulation assay of different CAR-T cells. (A, B) Flow cytometric analysis of degranulation levels of Control-T cells and CAR-T cells after incubation. **(C)** The data obtained for each group are compared in the histogram and shown as the mean ± SD (n=3). **$P < 0.01$, ***$P < 0.001$, and ****$P < 0.0001$.

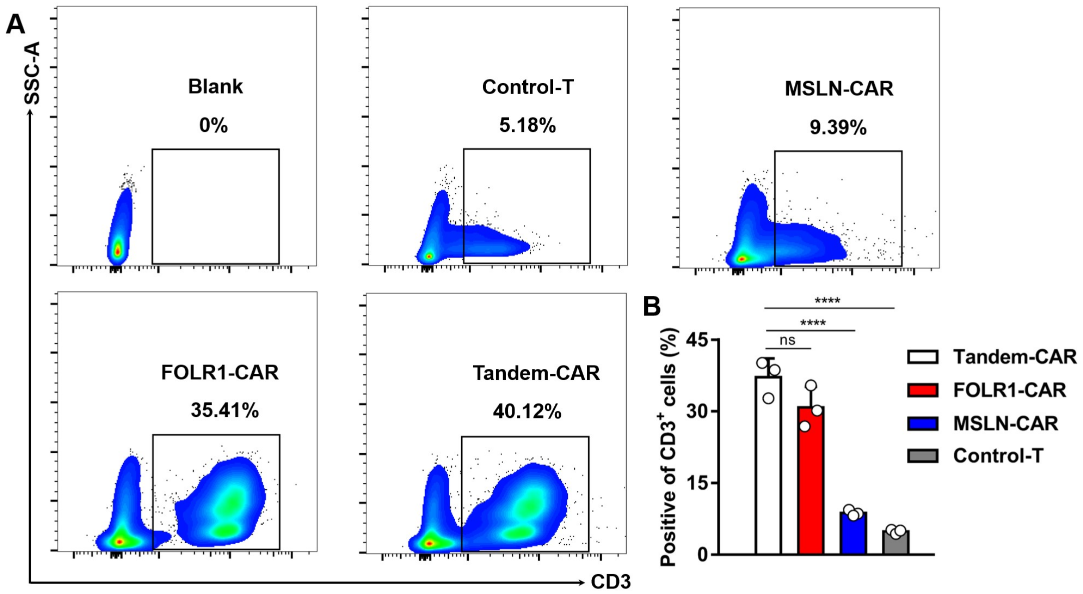

Figure S3. Persistence of Tandem CAR-T cells *in vivo*.

(A) The percentage of CD3-positive T cells in the peripheral blood was used to evaluate the expansion of CAR T cells. **(B)** The data obtained for each mouse are compared in the histogram and shown as the mean ± SD (n=3). ns: not significant and ****$P < 0.0001$.

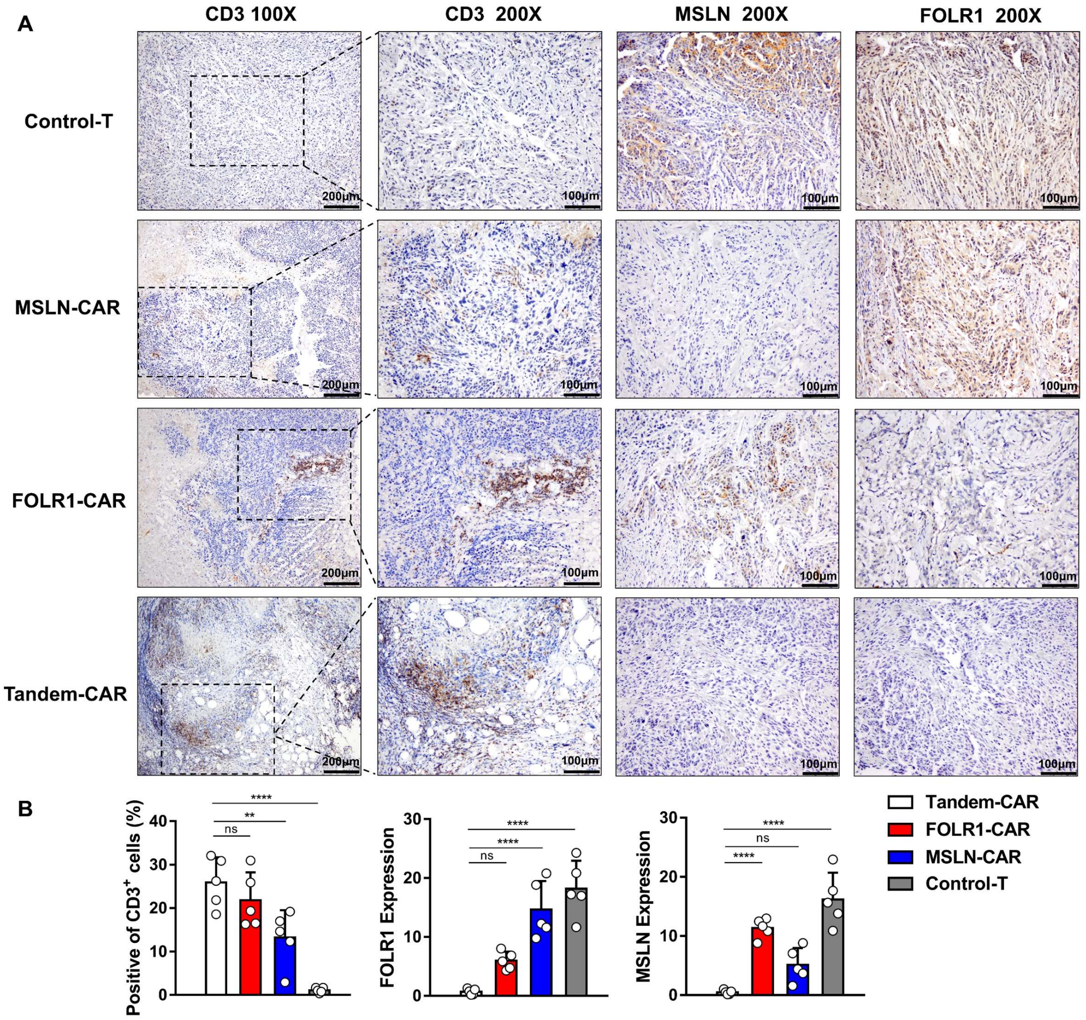

Figure S4. Measurement of T cell infiltration and antigen expression levels after CAR-T cell therapy.

(A) Representative photomicrographs of immunohistochemical staining. Paraffin tumor sections were stained with anti-human CD3ε, FOLR1, and MSLN antibodies. **(B)** The statistical analysis is represented in the histogram, and the data are shown as the mean ± SD (n=5). ns: not significant, $^{**}P < 0.01$, and $^{****}P < 0.0001$.

References

1. Tiraboschi C, Gentilini L, Velazquez C, Corapi E, Jaworski FM, Garcia Garcia JD, et al. Combining inhibition of galectin-3 with and before a therapeutic vaccination is critical for the prostate-tumor-free outcome. J Immunother Cancer. 2020;8(2).
